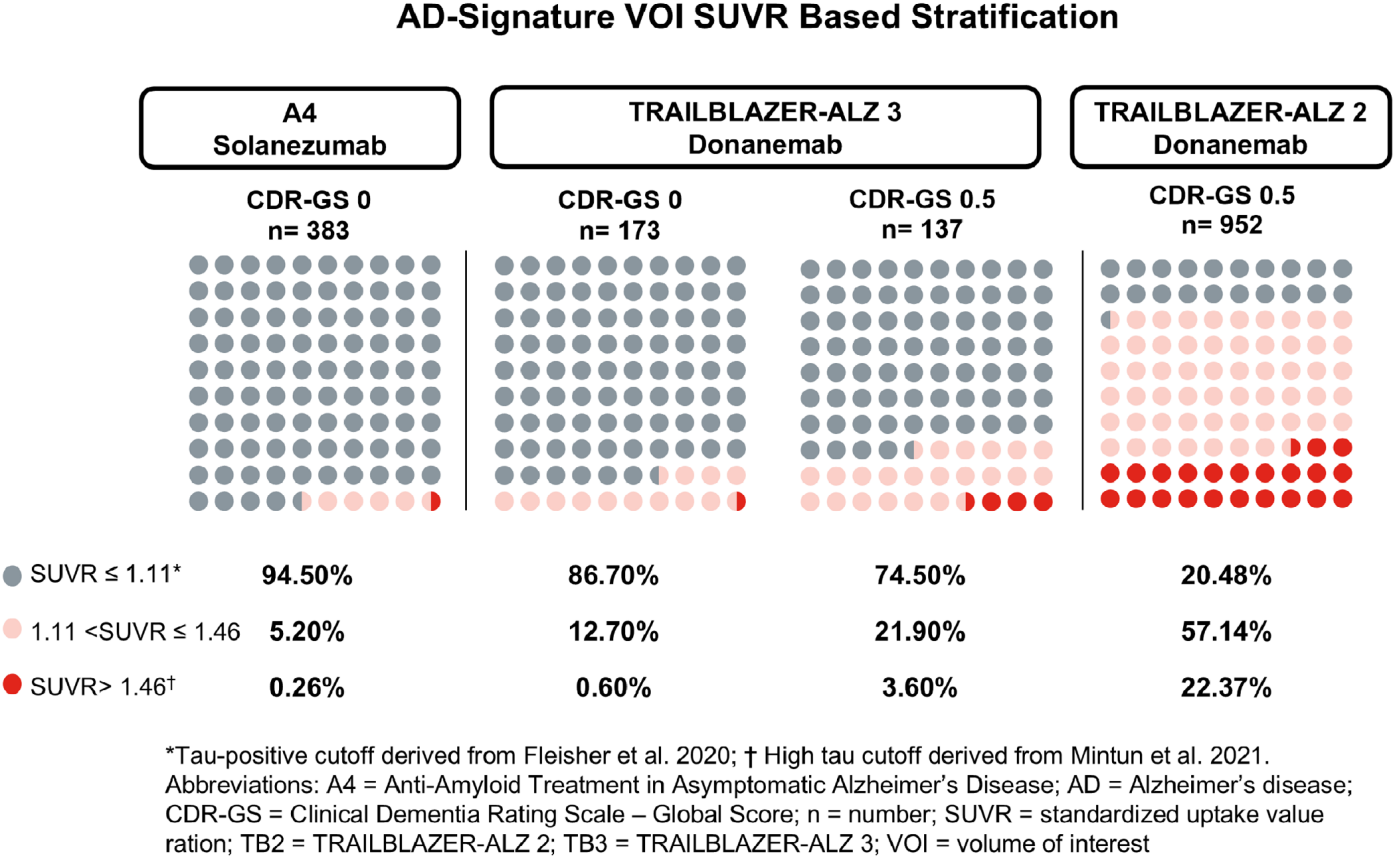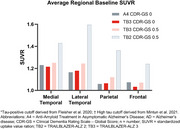# Flortaucipir patterns in preclinical Alzheimer's disease: an evaluation of TRAILBLAZER‐ALZ 3 baseline data

**DOI:** 10.1002/alz70856_101180

**Published:** 2025-12-25

**Authors:** Vikas Kotari, Karen Chilcott Holdridge, Roy Yaari, Melissa A M Williamson, John R. Sims, Sergey Shcherbinin

**Affiliations:** ^1^ Eli Lilly and Company, Indianapolis, IN, USA; ^2^ Eli Lilly & Company, Indianapolis, IN, USA

## Abstract

**Background:**

Preclinical Alzheimer's disease (AD) populations have evidence of elevated brain amyloid but are cognitively unimpaired. Most participants (95%) in solanezumab's preclinical AD trial, Anti‐Amyloid Treatment in Asymptomatic Alzheimer's Disease (A4), did not demonstrate elevated global tau accumulation at baseline. The ongoing TRAILBLAZER‐ALZ 3 trial will assess the efficacy of donanemab in preclinical AD participants. Here, we characterize and compare all available baseline tau positron emission tomography (PET) scans from TRAILBLAZER‐ALZ 3, A4, and the TRAILBLAZER‐ALZ 2 (early, symptomatic AD) study.

**Methods:**

TRAILBLAZER‐ALZ 3(NCT05026866) is a randomized, double‐blind, placebo‐controlled phase 3 trial. The study design was previously described. Baseline tau PET images were acquired using flortaucipir F 18 (FTP*)* as an optional sub‐study. Scans were analyzed using an established image processing pipeline to evaluate global tau burden and regional tau patterns. Global burden was measured using AD‐signature weighted neocortical standardized uptake value ratio (SUVR) relative to parametric estimate of reference signal intensity. Established SUVR cut points of >1.11 and >1.46 were utilized to quantitatively define positive FTP scan and high tau level, respectively. Regional tau patterns were assessed using multi‐regional tau staging schemes.

**Results:**

Baseline FTP scans were assessed for *N* = 310 participants (*n* = 173 with Clinical Dementia Rating‐Global Score, CDR‐GS 0 [CDR0] and *n* = 137 with CDR‐GS 0.5 [CDR0.5]). In the CDR0 and the CDR0.5 subgroups, 13.3% and 25.5% of participants, respectively, were tau positive (SUVR > 1.11; Figure 1). The medial and lateral temporal lobes had the highest regional baseline SUVRs for both subgroups. Average SUVRs for the CDR0 subgroup in medial temporal, lateral temporal, parietal, and frontal regions were 1.22, 1.18, 1.07, and 1.03, respectively, and 1.25, 1.24, 1.12, and 1.07 for the CDR0.5 subgroup, respectively (Figure 2).

**Conclusions:**

Although CDR0.5 subgroup participants demonstrated higher mean FTP regional SUVR values compared with CDR0 participants, the baseline tau PET patterns in TRAILBLAZER‐ALZ 3 are similar to other preclinical AD populations, even for participants in the CDR0.5 subgroup. Average regional baseline SUVR values support prior findings that tau deposition occurs sequentially from the medial temporal, lateral temporal, parietal, and frontal lobes. Additional regional analyses will be presented.